# ProcaryaSV: structural variation detection pipeline for bacterial genomes using short-read sequencing

**DOI:** 10.1186/s12859-024-05843-1

**Published:** 2024-07-09

**Authors:** Robin Jugas, Helena Vitkova

**Affiliations:** Department of Biomedical Engineering, Brno University of Technology, Brno, Czech Republic

**Keywords:** Copy number variation, Structural variation, CNV, SV, Bacteria, Pipeline

## Abstract

**Background:**

Structural variations play an important role in bacterial genomes. They can mediate genome adaptation quickly in response to the external environment and thus can also play a role in antibiotic resistance. The detection of structural variations in bacteria is challenging, and the recognition of even small rearrangements can be important. Even though most detection tools are aimed at and benchmarked on eukaryotic genomes, they can also be used on prokaryotic genomes. The key features of detection are the ability to detect small rearrangements and support haploid genomes. Because of the limiting performance of a single detection tool, combining the detection abilities of multiple tools can lead to more robust results. There are already available workflows for structural variation detection for long-reads technologies and for the detection of single-nucleotide variation and indels, both aimed at bacteria. Yet we are unaware of structural variations detection workflows for the short-reads sequencing platform. Motivated by this gap we created our workflow. Further, we were interested in increasing the detection performance and providing more robust results.

**Results:**

We developed an open-source bioinformatics pipeline, ProcaryaSV, for the detection of structural variations in bacterial isolates from paired-end short sequencing reads. Multiple tools, starting with quality control and trimming of sequencing data, alignment to the reference genome, and multiple structural variation detection tools, are integrated. All the partial results are then processed and merged with an in-house merging algorithm. Compared with a single detection approach, ProcaryaSV has improved detection performance and is a reproducible easy-to-use tool.

**Conclusions:**

The ProcaryaSV pipeline provides an integrative approach to structural variation detection from paired-end next-generation sequencing of bacterial samples. It can be easily installed and used on Linux machines. It is publicly available on GitHub at https://github.com/robinjugas/ProcaryaSV.

**Supplementary Information:**

The online version contains supplementary material available at 10.1186/s12859-024-05843-1.

## Background

Structural variation (SV) plays an important role in bacterial genomes [1–6]. They shape genome evolution, specialization, and adaptation. Genomic rearrangements can be reversible and temporarily beneficial, making bacterial genomes very plastic and adaptable to rapid changes. Short-term adaptive gene duplication can cause antibiotic resistance, which is an emerging issue [3]. Unlike large-scale SVs in eukaryotic genomes, large-scale SVs in bacteria are rare because of the constrained genomes of bacteria. The most common types of SVs present in bacteria are inversions, duplications, and insertions, often induced by horizontal gene transfer [3]. Deletions in bacteria are relevant to the topic of genome reduction and their implications. Gene loss by deletion was observed to surprisingly improve fitness and result in a faster growth rate [7]. A reduction in the genome density also increases genome stability and was first observed in symbiotic bacteria [8]. The molecular mechanisms underlying the occurrence of structural variations are similar in prokaryotes and eukaryotes [3]. Unlike eukaryotes, prokaryotic genomes have circular shapes, which further propagate into the symmetry of genome rearrangements [9, 10].

The majority of SV detection tools were designed and benchmarked on eukaryotic diploid genomes, and only a few were initially aimed at prokaryotes [11–13]. Despite that, they are successfully usable for detection in prokaryotic haploid genomes. The benefits of merging multiple independent tools have been successfully tested in eukaryotic genomes [14–16]. Recently, a pipeline for bacterial SV detection in long-read sequencing was published [17]. Also, a pipeline for SNV and indel detection for bacterial genomes exists [18]. Yet, an SV detection workflow using short-read sequencing reads for bacteria is missing.

In this study, we propose a pipeline named ProcaryaSV that integrates six SV and CNV detection tools based on reference alignment. These tools include CNproScan [13], CNVnator [19], LUMPY [20], DELLY2 [21], Pindel [22], and INSurVeyor [23]. These tools employ various approaches to detect four classes of SVs: deletions, duplications, inversions, and insertions. The selected tools were benchmarked before [13] and cover the whole spectrum of detection approaches. While these tools are commonly used, we added CNproScan, which is a tool that is able to detect very short CNVs present in bacterial genomes; these CNVs are generally more challenging to detect. Additionally, we added the recently published INSurVeyor to improve insertion detection [23].

## Implementation

### Pipeline design

**Fig. 1:**
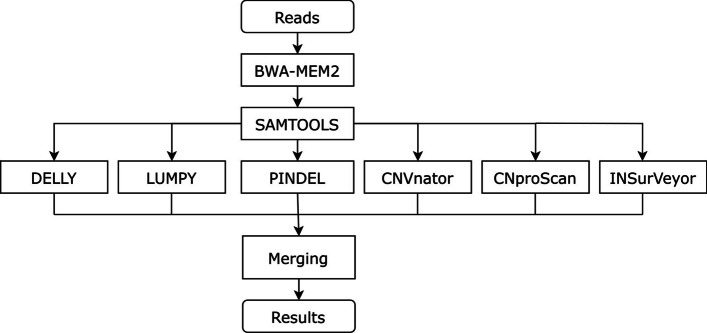
Simplified diagram of ProcaryaSV. The SV types are merged separately in the ProcaryaSV merging algorithm

The ProcaryaSV pipeline is implemented in the Python-based reproducible workflow Snakemake [24]. The workflow covers all the usual steps of sequencing data processing. A diagram of the pipeline is shown in Fig. 1. The process starts with a quality check of sequencing reads (FastQC [25]) and trimming (trim-galore [26]). The alignment is performed with BWA-MEM2, and BAM files are processed with SAMtools [27, 28]. Then, the BAM files serve as inputs for the SV and CNV callers. Additional inputs for detection tools are handled too, e.g., LUMPY requires separate BAM files for split and discordant reads. CNproScan uses the GenMap [29] output for mappability normalization. The complete list of tools and their versions is provided in the Supplementary Table, together with the Snakemake rules graph (Table 1).

**Table 1:** Overview of SV detection tools and SV types

SV type:	Being called by:
Deletions	DELLY2, LUMPY, PINDEL, CNVnator, CNProScan (5)
Duplications	DELLY2, LUMPY, PINDEL, CNVnator, CNProScan (5)
Inversions	PINDEL, DELLY2, LUMPY (3)
Insertions	PINDEL, DELLY2, LUMPY, INSurVeyor (4)

The outputs of SV callers are formatted as vcf files. These are subsequently input into ProcaryaSV’s merging algorithm. Additionally, we implemented the SURVIVOR merging algorithm as a reference tool for benchmarking [16, 30]. The final outputs are tab-separated files from ProcaryaSV’s merging algorithm and auxiliary plots. The pipeline computational requirements depend on a user-defined number of threads. We used a 12-core CPU, and the RAM usage reached approximately 10 GB in the artificial benchmarking.

### Merging algorithm

There are a limited number of tools available for merging structural variations. Some of the available tools often possess some limitations in terms of their use on haploid bacterial genomes [31–33]. We consider this field to be open to new inventions. Common approaches to SV merging include union or intersection, which are sometimes applied iteratively if multiple outputs are to be merged. The most common approach is the consensus approach. While merging two outputs is plain, merging multiple outputs is more challenging. The SV types were overlapped separately. A commonly used tool is part of the SURVIVOR toolkit [30], which we also employed in our pipeline. In SURVIVOR, two SVs are defined as overlapping if their start and stop coordinates are within 1 kbp and are of the same SV class. While SURVIVOR merging works with interval numeric operations, ProcaryaSV’s merging works based on signal processing.

Here, we demonstrate the new consensus-voting merging method based on signal summing. The genome rearrangements of each class and each caller are separately converted into a binary signal representation where a value of zero indicates the absence of the genome rearrangement and a value of one indicates its presence. The signal has the length of the reference genome used for alignment. The binary signals of SV callers for each SV type are summed together. In this signal, the value of three would denote a genome region called by three callers. Since it is a consensus merging process, the important parameter is the lower threshold, which determines which regions will be accepted. We call this parameter *minCallers*. The parameter *minCallers* was defined as the minimal number of callers supporting the presence of SV. Because we used six callers for the detection of deletions and duplications and four or three callers for the detection of inversions and insertions, respectively, we defined the *minCallers* parameter separately for CNVs, inversions, and insertions. These parameters can be set in the configuration file of the pipeline. Only events equal to or above this threshold are reported during postprocessing.

Multiplying the binary detection signal of the selected caller puts a higher weight on the caller. Generally, we do not employ weighting except for the insertion detection followed by the benchmarking. Thus, we doubled the weight of INSurVeyor in the detection. That means, that with *minCallers* of 2 for insertions, all events detected by INSurVeyor are detected. Alternatively, two other tools must call an insertion to be detected as positive.

**Fig. 2:**
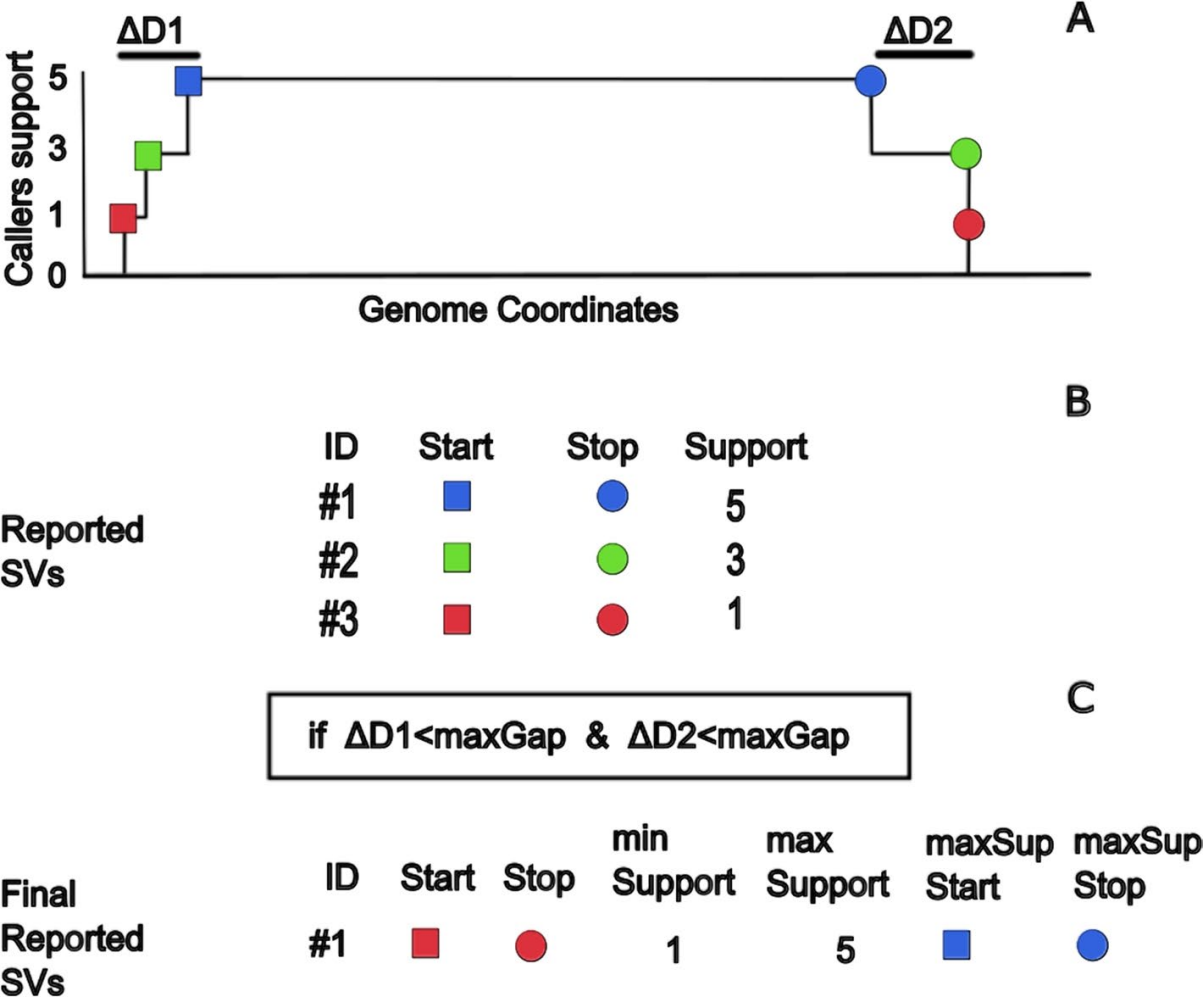
Overview of the SVs merging process. **A** The illustration of the SV signal and the effect of different start-stop coordinates by various callers. The colored dots indicate the values of start-stop coordinates. **B** The table of reported SVs from the example above. **C** The merged SVs if the start-stop coordinates are below the maxGap threshold for merging

During the merging, multiple overlapping reported SVs are created as a side effect. This process is graphically described in Fig. 2 and occurs because the coordinates of SVs reported by callers are not the same. The important parameter is user-defined *maxGap.* The maximum allowed distance between coordinates is defined such that the region is merged into one. If the distance between the corresponding start or stop coordinates reported by the original callers was greater than the *maxGap* value, the regions were reported as separate SVs. If the coordinates are in the range, we can use the information to report the narrowest and widest coordinates of a single SV. We call the narrowest coordinates the *maxSup start* and *stop* coordinates (see Fig. 2).

In certain cases, the SV signal consists of shorter peaks with a certain base value; e.g., the region was called one long rearrangement by two callers and multiple shorter rearrangements by another caller. In this case, depending on the *maxGap* value, there could be a single long SV with the support of 2 and multiple shorter SVs with greater support.

In the last step of the process of merging, the SVs are backtracked to the original calls reported by SV callers. This process serves to obtain the additional information reported by callers. Furthermore, the algorithms report the number of participating subevents together with their relative coverage. For example, an SV is covered by a certain number of smaller events called by a certain tool, and these events cover approximately 90% of the merged SV. The final result is formatted as a tab-separated (tsv) file. The merging algorithm is implemented as an R script and is called by the Snakemake workflow with user-defined parameters.

## Results

### Datasets

We created four artificial datasets to benchmark our pipeline. The first dataset is used to establish the optimal *minCallers* threshold values (*minCallers* dataset). The second dataset is used to validate these values and to benchmark the performance with other tools and pipelines (SV dataset). The third one has already been used in the past to benchmark CNV detection in bacterial genomes (CNV dataset) [12, 13]. Lastly, we evaluated the impact of GC content on detection. For overview see Supplementary Table.

The first two datasets benchmark all SV types separately. We randomly defined 100 SVs for each SV type, deletion, duplication, inversion, and insertion (400 SVs combined). The length of the SVs ranged from 50 to 10,000 bp. We used SVim to generate these SVs [34]. Because SVsim simulates insertions by taking them from the other chromosomes in the input FASTA file, we have to use our in-house script to create artificial insertions. We benchmarked these coverage values as follows: 5 × , 10 × , 20 × , 50 × , and 100 × . The minCallers dataset uses *Escherichia coli* str. K-12 substr. MDS42 (GenBank: AP012306.1) as source of sequencing reads. We benchmarked insertions from another different bacteria and from another strain of *E. coli* to see how detection performs based on the origin of insertions sequence (see Supplementary Table for sequence details). The read length was 150 bp.

The SV dataset was created in the *K. pneumoniae* genome subsp. pneumoniae NTUH-K2044 (GenBank: NC_012731), and artificial insertions were inserted from the *S. aureus* subsp. aureus USA300_FPR3757 genome (GenBank: NC_007793.1). The read length was 75 bp.

The third CNV dataset consists of 30 artificial deletions and duplications of various short lengths and copy numbers imputed into the genome of *Staphylococcus aureus subsp. aureus* TW20 (GenBank: NC_017331). We benchmarked four coverage values: 5 × , 10 × , 20 × , 100 × , and 200 × . The read length was 75 bp. Artificial reads for all datasets were generated with art-sim [35].

The real samples dataset contains sequencing data for bacterial isolates from three projects, which are listed in Supplementary Table [36–38]. In these datasets, there were 190 bacterial isolate samples sequenced with short reads with an average coverage of 60 × to 350 × .

Artificial benchmarking involves resolving several aspects. First, efficient *minCallers* values are set based on the performance metrics. Second, we measured the performance of ProcaryaSV’s merging algorithm against the SURVIVOR merging algorithm. Third, the performance of the whole pipeline was compared to that of the Parliament2 pipeline. Lastly, we can see how merging improves the detection compared to individual callers.

### Defining optimal minCallers values

We employed the first dataset to obtain ideal values of minimal consensus threshold called *minCallers* in our pipeline. The *minCallers* values are defined independently for CNVs, inversions, and insertions.

We assessed the optimal value of the *minCallers* parameters with the use of precision and recall values (see Supplementary Table). We ran the pipeline multiple times with different *minCallers* values being set. Then, we calculated performance scores for all the *minCallers* and coverage levels.

For CNVs, defining exact *minCallers* values is not straightforward. The range of values from 2 to 4 seems to be optimal for coverage above 20 × . For lower coverage, setting *minCallers* to 2 or 3 is optimal. We repeated the analysis of optimal *minCallers* in the CNV dataset, which includes very small CNVs. Here, we discovered that the *minCallers* set to 2 achieved the highest accuracy and F1 scores across all coverage levels. Many false positives were detected for the *minCallers* set to 1, caused by Pindel and CNproScan, and were eliminated by increasing the value to 2. On the other hand, Pindel and CNproScan were able to detect the shortest CNVs. Observing the precision-recall curves (see Supplementary Table), we conclude that the optimal value of *minCallers* is 2 for a broad range of CNV lengths, including small lengths. For generally longer CNVs, which are easier to detect, the *minCallers* can be set to a value of 3 or 4.

For inversions, the optimal *minCallers* value is 2. As inversions can be detected by three detection tools at most, the choice of 2 was straightforward. Also, the detection of inversions is not coverage-dependent.

Insertions are most effectively called by INSurVeyor and three other tools. Since the elevated weight for INSurVeyor, the values 1 and 2 perform similarly. Furthermore, we compared the performance of insertion detection with distant and more similar sequences. Insertions of taxonomically close origin (different strain of bacteria in this case) are more challenging to detect.

### Artificial CNV dataset

We used the CNV dataset to evaluate the performance of ProcaryaSV for detecting small CNVs and to estimate the optimal value of the *minCallers* parameter for small CNV detection (see previous section). The complete results and plots are provided in the Supplementary Table.

Second, we compared ProcaryaSV’s merging algorithm with the SURVIVOR merging algorithm. SURVIVOR was chosen because of its easy implementation and usability with selected callers. Additionally, we tested the SVDB merging tool [39]; however, the output was not reliable for use because the SVDB merging tool inserts modified sequences into the vcf file. The SURVIVOR merge settings for minimal callers were set to 2, the maximum allowed distance was set to 1000, and the minimal considered SV length was set to 1. The *minCallers* parameter of ProcaryaSV was also set to 2. We achieved similar results, as shown in Table 2. The values in bold signify the highest values. The ProcaryaSV had higher accuracy and F1 scores for 20 × and higher coverage by a few percent. Generally, the results are comparable to what we expected. The results reflect the congruency between the methods.

**Table 2:** Results of the artificial CNV dataset (ProcaryaSV *minCallers* 2)

	Coverage	Accuracy	Sensitivity	Precision	Specificity	F1 score
ProcaryaSV	5 ×	**90.0**	**80.0**	**100.0**	**100.0**	**88.8**
	10 ×	90.0	80.0	100.0	100.0	**88.9**
	20 ×	**90.0**	**80.0**	100.0	100.0	**88.9**
	100 ×	**87.3**	**83.3**	89.3	90.9	**86.2**
	200 ×	**88.9**	**86.7**	89.7	90.9	**88.1**
SURVIVOR	5 ×	88.5	80.0	96.0	96.8	87.3
	10 ×	90.0	80.0	100.0	100.0	88.9
	20 ×	85.0	70.0	100.0	100.0	82.4
	100 ×	87.1	80.0	92.3	93.8	85.7
	200 ×	85.5	76.7	92.0	93.8	83.6
Parliament2	5 ×	78.3	56.7	100.0	100.0	72.3
	10 ×	78.3	56.7	100.0	100.0	72.3
	20 ×	83.3	66.7	100.0	100.0	80.0
	100 ×	83.3	66.7	**100.0**	**100.0**	80.0
	200 ×	83.3	66.7	**100.0**	**100.0**	80.0

Third, we compared the performance against the Parliament2 pipeline. We used Parliament2 with Breakdancer[40], CNVnator[19], DELLY2[21], Manta[41], and LUMPY[20]. Merging in Parliament2 is performed natively with SURVIVOR[30]. We put the results into Table 2. The first notion is that Parliament2 results are less coverage independent. However, we achieved higher scores except for precision and specificity at 100 × and 200 × coverage.

Finally, we analyzed the redundant tools via UpSet plots [42]. These plots are in the Supplementary Table. Considering only CNVs, the CNVnator detected the least number of CNVs. None of them were detected uniquely by the CNVnator. On the other hand, it participated in the detection of some low-coverage events.

We also evaluated individual callers separately to see how the SV merging improved overall detection (see Supplementary Table). Regarding the F1 scores, the DELLY2 and LUMPY are the best-performing tools across all the coverage levels. In sensitivity, the CNproScan detects the highest number of true positives. Generally, the merging of small-sized CNVs brought performance benefits.

### Artificial SV dataset

We benchmarked the performance also on the validation SV dataset and compared it again with SURVIVOR, Parliament2, and independent tools. We set the *minCallers* threshold for all SV types to 2 but also included a value of 3 for CNVs. The F1 scores of the competing methods are shown in Table 3. For the rest of the metrics and plots, see Supplementary Table.

**Table 3:** F1 scores of the artificial SV dataset (ProcaryaSV minCallersCNV = 2, minCallersINV = 2, minCallersINS = 2)

	Coverage	Deletions	Duplications	Inversions	Insertions
ProcaryaSV	5 ×	97.1	**98.5**	**100.0**	**27.4**
	10 ×	**100.0**	**99.0**	**99.5**	**65.1**
	20 ×	**100.0**	**99.5**	99.5	**77.2**
	50 ×	**99.5**	**99.5**	98.0	**76.5**
	100 ×	**98.0**	98.0	95.7	59.3
SURVIVOR	5 ×	83.5	80.2	80.6	0.0
	10 ×	99.5	99.0	97.1	3.8
	20 ×	99.0	99.5	100.0	13.1
	50 ×	97.5	98.0	99.0	12.7
	100 ×	95.1	98.0	97.1	10.7
Parliament2	5 ×	**98.0**	**98.5**	87.0	0.0
	10 ×	98.5	**99.0**	98.5	1.9
	20 ×	98.5	**99.5**	**100.0**	8.6
	50 ×	97.4	**99.5**	**100.0**	11.8
	100 ×	97.4	99.5	**100.0**	13.1

The performance for deletions and duplications was stable across different coverage values. The detection of large CNVs is not as dependent on coverage as the detection of short CNVs is. The inversion results were also stable, with a small decrease toward high coverage. This was caused by 5 inversions misclassified as duplications.

The most challenging part was the detection of insertions. This is attributed to the nature of short-read sequencing. In the evaluation, we increased the boundaries of the exact breakpoints by 50 bp so that we could match the breakpoints with the detected insertions. Of the four tools used to detect insertions, three can detect only short insertions via split-read alignment (Pindel, LUMPY, DELLY2). This requires that the length of the insertion fit into a single read length [20–22]. Furthermore, detection is difficult when the insertion is similar to regions in the reference genome. The employed INSurVeyor uses read-pair and de novo assembly methods to detect a larger scope of insertions [23]. INSurVeyor is responsible for a major boost in insertion detection against competitors.

As previously described, the merging results are comparable between the two implemented algorithms, ProcaryaSV’s and SURVIVOR’s merging methods. The differences are small, benefitting the first method by a few points.

ProcaryaSV slightly outperformed Parliament2 in terms of deletions and largely in detection insertions. The Parliament2 was slightly better in inversions. Both pipelines performed similarly in duplications. The performance of Parliament2 matched the performance of ProcaryaSV when *minCallers* were set to 2.

DELLY2 and LUMPY, as in the CNV dataset, are very well-performing tools in the detection of deletions, duplications, and inversions. Unlike in CNV dataset results with short CNVs and indels, the performance of these two individual tools is comparable with the merging approach. However, when we observed the results, we noticed that individual tools tend to call multiple shorter events along the original long SV. All these are detected as true positives in our case (as they overlap with defined intervals), but the merging method overcomes this drawback of individual callers and merges them into one continuous event.

Finally, we observed the performance of each tool via UpSet plots of the true-positive SVs (see Supplementary Table). Unlike for short CNVs, the least well-performing tool for CNV detection was Pindel. The majority of events were detected by the other tools. Pindel was more useful for detecting inversions, yet a large share of detected inversions were also called by other tools. In contrast, Pindel is indispensable for insertion detection. Most of the insertions were detected with INSurVeyor seconded by Pindel.

### GC content impact on detection

Lastly, we evaluated the impact of GC content on detection. We benchmarked simulated CNV datasets of three different bacteria, representing low, middle, and high GC content. The selected bacteria were *Staphylococcus aureus* (GC 33%), *Klebsiella pneumoniae* (GC 57%), and *Anaeromyxobacter dehalogenans* (GC 74%). The details about sequences and dataset creation are in the Supplementary Table, although the same recipe as in the SV dataset was used.

The detection results correspond to the previous results. The lowest and the highest coverage is slightly the most challenging. However, we found no performance impact associated with different GC content, as can be verified in the Supplementary Table.

### Real dataset

Benchmarking on real data was performed to assess the usability of the pipeline for real data. Since no apriori-defined SVs are known, the space for evaluation is limited. We can conclude the overlap between various tools and features of detected SVs depending on the SV class and the caller. The results are provided in the Supplementary Table. All samples of the same species were pooled together in the final analysis.

All SV types were detected in samples of *K. pneumoniae*. The majority of inversions were called by DELLY2 and Pindel, unlike the combination of DELLY and LUMPY in the artificial dataset. The insertion results copied those of artificial ones, with DELLY2 detecting a significant portion of the insertions. Interestingly, only two duplications were detected by all five tools. The number of detected deletions was much greater. The *L. casei* samples had the lowest number of SVs. No insertions were detected. In contrast, *S. aureus* had high numbers of SVs called by multiple tools. There were 60 insertions called by both Pindel and INSurVeyor, and the number of inversions called by the three callers was also high.

Despite that we cannot point to the accuracy of the real dataset detection, it is interesting to see the differences in detection compared to artificial datasets. While DELLY and LUMPY performed well as individual CNV and inversion callers on the artificial SV dataset, they each detected a distinct set of SVs. Here, we see fully the benefit of the merging approach.

### Computational performance of ProcaryaSV

Data analysis of bacterial genomes, which are only megabases in length, is not a computationally demanding task given modern PC specifications. The pipeline was tested on a 12-thread CPU with 64 GB of RAM. The run times reported by the Snakemake workflow are in the Supplementary Table. The RAM usage generally did not exceed 10 GBs when 12 threads were used.

## Discussion

We developed a consensus-based pipeline for structural variation detection in bacterial genomes sequenced via short-read technologies. Although long-read sequencing, mainly nanopore sequencing, has become widely used in bacterial sequencing, short-read sequencing is still widely used, and with the arrival of new vendors in the field, the costs of sequencing will likely further decrease [43].

While many SV detection tools and pipelines have been presented, not many were tested on bacterial genomes. There is a long-read SV detection pipeline [17] and a short-read SNV and indel pipeline [18], but there is a gap in the bacterial SV detection pipelines.

We compared our pipeline with Parliament2 [16]. Like in Parliament2, we also implemented the SURVIVOR merging algorithm [30]. We developed our method for SV merging and tested it against SURVIVOR. The two merging methods are comparable in their results; in certain cases, our merging algorithm provides better results. Most importantly, our method enabled us to tweak the so-called *minCallers* parameter defining the minimal callers' support to call an SV. We defined this parameter separately for CNVs, inversions, and insertions for the best performance.

Comparing the performance of the two pipelines for SV detection, ProcaryaSV, and Parliament2, the former produced better results across the whole dataset and drastically better results in insertion detection. Unlike Parliament2, our pipeline is a complete workflow that includes read trimming and alignment. Parliament2 requires an aligned BAM file. Additionally, ProcaryaSV is implemented in the popular Snakemake workflow, which is easy to configure, use, and modify ad hoc. Snakemake workflows are highly scalable in terms of performance.

An important step was the selection of tools to include in the pipeline. We used tools we had previously successfully tested [13], and they are also commonly used. We also included recently published INSurVeyor aimed at insertion detection. Therefore, we were able to present results that outperformed those of the competition. Some tools could be skipped, and this can be performed easily by the user via a configuration file. CNVnator and Pindel could be two candidates for exclusion. Pindel detects several false positives but is useful for insertion detection. CNVnator participated modestly in the CNV dataset. However, when observing the UpSet plots, we believe that they are still usable and increase the robustness of the consensus voting-based approach.

We see the potential of the merging approach when observing the discordance between the results of artificial and real datasets in the overlap diagrams. Unlike in the *in-silico* data, detection tools detect distinct sets of structural variations. These differences point out the benefits of the merging approach.

General limitations of SV detection originate from the fact that we are inferring them from indirect signatures in the alignment data and that SVs are longer than the size of sequencing reads. Limitations we are specifically aware of are the insertion detection and merging algorithm designed for short genomes. Three callers out of four can detect only insertions fitting into the read length. Only INSurVeyor can detect larger ones. Furthermore, the insertion detection is limited by the genomic origin of the insertion itself with genetically closer insertions being more challenging to detect. The ProcaryaSV’s merging algorithm was designed with bacteria-size genomes in mind and will take more computational time if the genome size increases beyond the usual size of several millions of bases. This is because of the signal representation of the reference genome.

## Conclusions

In this study, we presented a ProcarySV, an SV/CNV detection workflow focused on bacterial research. We implemented a total of six tools to increase the performance metrics and to find the most accurate genome rearrangements. We also wanted to provide an easy-to-use workflow, which demands a certain kind of bioinformatics knowledge, yet saves time by studying the specifics of each detection tool and computing the tailored inputs for some callers.

The essential task for acquiring high-accuracy results is the robust merging of genome rearrangements. Therefore, we also presented a novel merging algorithm based on a signal representation of detected events. This algorithm is suitable mainly for bacterial genomes because of their small size and the occurrence of small structural variations. It is also effective at merging multiple detection tools.

The pipeline covers the whole workflow beginning with the processing of sequencing reads, alignment, quality reports, and SV detection, ending with the final list of detected SVs.

### Supplementary Information

Supplementary Material 1: Table Overview of the results, information about the dataset, and plots.

## Data Availability

The ProcaryaSV homepage and code can be found at https://github.com/robinjugas/ProcaryaSV. The datasets used are stored at 10.5281/zenodo.11552616.

## Availability and requirements

Project name: ProcaryaSV, Project home page: https://github.com/robinjugas/ProcaryaSV, Operating system(s): Linux, Programming language: Python3, R, Bash, and Snakemake, Other requirements: Python 3.6 or higher, Snakemake 5.13 or higher, License: MIT license, Any restrictions to use by non-academics: none.
